# Recent advances in treatment of severe primary immunodeficiencies

**DOI:** 10.12688/f1000research.7013.1

**Published:** 2015-12-16

**Authors:** Andrew Gennery

**Affiliations:** 1 Paediatric Immunology and Haematopoietic Stem Cell Transplantation, Institute of Cellular Medicine, Newcastle University, Newcastle upon Tyne, UK; 2 Paediatric Immunology and Haematopoietic Stem Cell Transplantation, Great North Childrens’ Hospital, Newcastle upon Tyne, UK

**Keywords:** immunodeficiency, Primary immunodeficiency, hematopoietic stem cell, hematopoietic stem cell transplantation, Haematopoietic Stem Cell, Haematopoietic Stem Cell Transplantation, Severe combined immunodeficiencies, Chemotherapy conditioning

## Abstract

Primary immunodeficiencies are rare, inborn errors that result in impaired, disordered or uncontrolled immune responses. Whilst symptomatic and prophylactic treatment is available, hematopoietic stem cell transplantation is an option for many diseases, leading to cure of the immunodeficiency and establishing normal physical and psychological health. Newborn screening for some diseases, whilst improving outcomes, is focusing research on safer and less toxic treatment strategies, which result in durable and sustainable immune function without adverse effects. New conditioning regimens have reduced the risk of hematopoietic stem cell transplantation, and new methods of manipulating stem cell sources should guarantee a donor for almost all patients. Whilst incremental enhancements in transplantation technique have gradually improved survival outcomes over time, some of these new applications are likely to radically alter our approach to treating primary immunodeficiencies.

## Introduction

Genetically inherited inborn errors of immunity impair immune function, which leaves affected individuals exposed to increased risks of infection, inflammation and autoimmunity. To date, over 300 diseases with X-linked, autosomal recessive and autosomal dominant inheritance have been identified ^1^. The majority of described diseases result from complete or partial loss of function of the gene product, but more recently, increasing numbers of diseases in which the gene mutation leads to a gain-of-function effect have been described. For an increasing number of diseases, replacement of the defective recipient immune system with a functioning system from a healthy donor, by hematopoietic stem cell transplantation (HSCT), can lead to a permanent cure.

The first HSCTs for primary immunodeficiencies (PIDs) were performed in 1968 ^2, 3^, and so nearly 50 years of experience has led to many significant improvements in technique and outcome. Severe combined immunodeficiencies (SCIDs) are the most profound defects, and HSCT, until recently, has been the only approach to treatment (with the exception of adenosine deaminase [ADA] deficiency, for which enzyme replacement is possible). Other PIDs have had conservative or HSCT approaches to management, although HSCT is now becoming a more widely accepted modality of treatment, as long-term outcomes of conservative management are investigated, and outcomes improve through earlier diagnosis and safer approaches to transplantation.

Treatment of PIDs has resulted in the recognition of better outcomes with early or pre-emptive treatment, development of newborn screening programs for PID, development of gene therapy, and is now driving the development of gene editing as well as the search for minimally toxic conditioning regimens. This article will outline recent developments in the field.

## Severe combined immunodeficiencies

SCIDs are heterogeneous PIDs that are characterized by the absence of thymopoiesis, T-lymphocyte maturation and function, and which affect cellular and humoral acquired immunity; without definitive treatment within the first 12 to 18 months of life, the condition is invariably fatal. Classic presentation is with persistent viral respiratory or gastrointestinal infection in infancy and with failure to clear virus and persistent and deteriorating symptoms ^4^. Multiple pathogens may co-exist, and opportunistic infection—for example, with *Pneumocystis jiroveci*—is common. Immunization with live Bacillus Calmette-Guerin or rotavirus vaccine can cause persistent and disseminated infection ^5– 7^. The genetic bases of 75% to 80% of SCID types are now understood. Definitive treatment is predominantly by allogeneic HSCT, although gene therapy and enzyme replacement therapy are available for some specific genetic sub-types. Depending on the genetic defect, recipient B-lymphocyte or natural killer (NK) cells or both may be present. In contradistinction to the treatment of hematological malignancies, in which eradication of malignancy is required, the objective of HSCT in patients with SCID is to provide normal HSCs, facilitating correction of the immune defect. Therefore, it is critical to minimize potential sequelae of treatment but to establish effective long-term immune function. The outcome of HSCT for SCID is related to a number of different factors, including genotype, pre-existing morbidities at time of HSCT, and in particular pre-existing viral infection, as well as the type (and degree of human leukocyte antigen [HLA] match) of the donor ^8– 10^. Current issues of interest to address include early detection of infants with SCID, so that referral for treatment may be initiated before the onset of infection, and approach to conditioning.

## Newborn screening for severe combined immunodeficiency

During T-lymphocyte receptor development, redundant DNA is excised but remains within the cell and can be used as a marker of thymopoiesis. Patients with SCID (and some other PIDs) lack thymopoiesis and subsequently the redundant DNA (known as a T-cell receptor excision circle, or TREC) is not present. With the blood taken during routine neonatal screening, it is possible to detect TRECs by polymerase chain reaction and thus identify infants with SCID before symptoms develop ^11, 12^. Alternately, for two related PIDs in which a DNA salvage enzyme is deficient, leading to absent lymphocyte development (ADA and purine nucleoside phosphorylase deficiency), metabolic by-products can be detected by mass spectrometry on blood eluted from the neonatal blood spot ^13, 14^. It has previously been demonstrated that the outcome of HSCT for newborn patients with SCID is significantly superior to that of patients presenting with infection ^8– 10^. The introduction of newborn screening enables the detection of infants with SCID before they become symptomatic, allowing definitive treatment before they acquire infection. Many states in the USA have now implemented newborn screening for SCID ^15^, enabling early detection and treatment. Other countries are considering implementation of newborn screening programs for SCID and other PIDs ^16, 17^.

## Chemotherapy conditioning

Following appropriate diagnosis, there remains debate about the best approach to treatment. Infusion of unfractionated donor HSC inoculum without preparative chemotherapy leads to T-lymphocyte immune reconstitution ^18^. However, without HSC engraftment, the establishment of thymopoiesis and the durability of T-lymphocyte function are variable and depend on the phenotype and hence genotype. Infants with NK cell-negative SCID are more likely to survive than those who have recipient NK cells, and also develop high-level donor T-lymphocyte chimerism with superior long-term persistence of CD4 ^+^ T-lymphocyte immunity without preconditioning chemotherapy. The presence of recipient NK cells is a strong indicator that preparative chemotherapy conditioning will be required for engraftment of T-lymphocyte precursors capable of supporting robust and durable T-lymphocyte reconstitution ^19^. However, the type of donor used is also important, as use of an unrelated HLA-matched donor, rather than an HLA-matched sibling donor, significantly increases the risk of graft-versus-host disease (GvHD) ^20^.

There are a number of issues regarding the use of chemotherapy preconditioning. Acute toxicities are frequently observed, and in the presence of active infection, mortality is increased unless a matched sibling donor is available ^10^. Whilst durability and sustainability of thymopoiesis and consistency of B-lymphocyte function are more likely in most forms of SCID following chemotherapy and, in particular, those genotypes with recipient NK cells ^19, 21, 22^, there are concerns about the effects, albeit short courses, of chemotherapy on young infants. There are currently no good multicenter studies looking at the long-term (>20 year) immunological, general health or quality-of-life outcomes of HSCT in SCID, either using chemotherapy preconditioning or just infusing the donor inoculum. A joint European Inborn Errors Working Party/North American Primary Immune Deficiency Treatment Consortium study looking at these outcomes is in progress. However, for a subset of patients with radiosensitive SCID, alkylating agents are associated with more significant long-term co-morbidities, even when compared with other NK cell-positive SCID phenotypes ^23^. New conditioning regimens with analogues of busulfan appear safer and have fewer short-term toxicities, but long-term outcomes are uncertain ^24, 25^. The problems of administering chemotherapy will become more focused only as the majority of infants begin to be diagnosed in the newborn period through newborn screening programs. Whilst the mortality risk associated with chemotherapy, particularly in well patients with no co-morbidities, is low, it is not absent, and the concerns over administering such toxic drugs to newborns are driving the search for alternative strategies. Minimally intensive regimens using monoclonal antibodies have been successfully employed in treating SCID, even with significant co-morbidities, but these still employ low-dose chemotherapeutic agents ^26^. Unfortunately, to date, neither treatment with alemtuzumab monotherapy nor plerixafor in conjunction with granulocyte-colony-stimulating factor appears to facilitate donor stem engraftment in patients ^27, 28^. *In utero* animal models have demonstrated some beneficial effect of administering an anti-c-Kit receptor antibody, which interrupts an important signaling pathway in homing, adhesion, maintenance, and survival of HSCs in the hematopoietic niche, and transplanting pre-treated animals on the first day of life; some gain in donor stem cell engraftment was observed ^29^. Whilst these results are encouraging, further work is required before patient benefit can be demonstrated. However, clinical trials using therapeutic-grade antibodies are being planned.

## Gene therapy

Gene therapy for SCID has been successful at curing patients. Random integration of a viral vector containing the corrected gene into the genome of harvested autologous HSCs and re-infusion of the transduced product have demonstrated clinical benefit and cure of patients with X-linked and ADA-deficient SCID ^30, 31^. Early trials were complicated by graft failure and in some cases insertional mutagenesis, leading to lymphoproliferation and leukemia, at least in X-linked SCID ^32, 33^. Modifications of the retroviral vector with the addition of self-inactivating gamma-retroviral vectors, with enhancer-deleted U3 regions ^34, 35^, and adoption of lenti-viral vectors should reduce or eliminate the risk of insertional mutagenesis ^36, 37^. New methodologies of gene editing use highly specific, targeted double-stranded DNA cleavage nucleases to remove the defective gene and replace it with a corrected copy at the appropriate genomic locus through the use of homologous recombination of corrected gene sequences by cellular DNA repair pathways ^38– 40^. These techniques are a more physiologically sound method of genetic correction as the appropriate regulatory control of gene expression is maintained.

Pre-clinical studies have demonstrated efficacy of this technique in cell lines, although correction in primary HSCs has been limited. Current gene therapy protocols do not consistently result in full correction of the defect, and in some trials, low-dose chemotherapy has been employed to improve autologous stem cell engraftment and give a competitive advantage over non-transduced cells. Thus, in an approaching era of hopefully universal screening for SCID, a chemotherapy-free approach for either conventional HSCT or gene-targeted therapy may be possible, eliminating concerns about long-term toxicity and ensuring durable and sustainable immune reconstitution.

## Alternative treatments for severe combined immunodeficiencies

For a few SCID genotypes, alternative therapies are available and, though not curative, may improve the physical condition of the patient pending curative treatment. For patients with ADA-deficient SCID, polyethylene-glycosylated adenosine deaminase (PEG-ADA) can be given as an infusion, thus partially reversing the enzyme deficiency. This can rapidly reverse some of the toxicity associated with ADA deficiency (for example, ADA deficiency-related pulmonary alveolar proteinosis ^41^) and improve the clinical condition of the patient to facilitate successful HSCT ^42^. Long-term treatment with PEG-ADA leads to poorer immunoreconstitution than following HSCT ^43^ and gene therapy and may induce PEG-ADA-specific antibody formation, compromising further immunoreconstitution ^44^.

Defects in the folate and cobalamin pathway can impact immune development ^45– 47^. Recently, a patient with SCID immunophenotype has been described ^48, 49^, in whom a mutation in *MTHFD1*, which encodes a protein essential for folate metabolism, was found. Treatment with folate and hydroxocobalamin improved but did not fully correct lymphocyte counts and proliferation responses, although no benefit to the neurological impairment was observed ^48– 50^.

Phosphoglucomutase 3 (PGM3) is a hexose phosphate mutase, a key enzyme in many glycosylation pathways. Mutations in *PGM3* are associated with neutropenia, B and T lymphocytopenia and bone marrow failure ^51, 52^, although extra-immune manifestations, including facial dysmorphism, skeletal anomalies and intellectual impairment, are also apparent. Conventional HSCT cures the immunological features but not the other features. N-acetyl-galactosamine supplementation may have a role in bypassing the metabolic defect and stabilising these patients prior to definitive treatment. Thymic stromal defects—for example, complete DiGeorge or CHARGE (coloboma, heart defect, atresia choanae, retarded growth and development, genital hypoplasia, ear anomalies/deafness) syndrome or FOXN1 deficiency—can be successfully treated by thymic transplant and with better outcomes than HSCT ^53– 55^.

## Other primary immunodeficiencies

An increasing number of non-SCID PIDs are successfully treated by HSCT. A patient with Wiskott-Aldrich syndrome was among the first to undergo HSCT ^2^. Subsequently, HSCT has been performed for many different PID diseases. HSCT initially was restricted to patients with combined immunodeficiencies or severe T-lymphocyte defects, but now there is an expanding list of appropriate indications. Specifically, cytotoxicity defects (e.g., familial hemophagocytic syndromes), defects of phagocytes (e.g., chronic granulomatous disease, leukocyte adhesion deficiency, and GATA2 deficiency), and defects in cytokine signaling pathways are now indications for HSCT. As the definition of immunodeficiency broadens, so do the indications for HSCT ^56^. Patients with autoimmune disease or autoimmune enteropathy, including those with immunodysregulation, polyendocrinopathy, enteropathy, X-linked syndrome, defects in interleukin-10 (IL-10) signaling pathways, gain-of-function STAT-3 disease, and CTLA-4 deficiency, have received curative transplants. Often patients may achieve a molecular diagnosis following successful transplantation, so proof of concept of treatment may precede genetic diagnosis.

Patients with non-SCID PID have residual T lymphocyte-mediated immunity which is able to mediate rejection of allogeneic grafts, necessitating pre-transplant chemotherapy conditioning to facilitate donor stem cell engraftment and immune reconstitution. The decision to refer for HSCT can be difficult, as many diseases have alternative medical therapies, which if taken regularly may prevent many complications from developing. However, careful cohort studies indicate that, even with regular medication, life-threatening complications occur, and quality of life may be significantly and adversely affected. With modern HSCT techniques, the survival outcome following HSCT may be equivalent to “conventional” treatment ^57^. In addition, successful HSCT for non-SCID PID may lead to an improved, and even normal, quality of life, as well as abolition of the risk of disease-associated sequelae, and removal of necessity to take life-long medication ^58^. Unfortunately, as for patients with SCID, outcomes are best for those with no pre-existing co-morbidities ^59, 60^. Parents may be left with the choice of transplanting a young healthy child, and accepting the small but finite risk of failure and likely death, or waiting until the child is more sick with established co-morbidities but with a diminished chance of successful transplantation. For some patients with non-SCID PID, immunodeficiency is part of a wider syndrome. Successful HSCT can correct the hemato-immunological defect, but extra-immune manifestations are generally not modified ^61^ and may present many years after successful HSCT ^62^.

## Safer chemotherapy conditioning regimens

Several recent advances are changing the landscape for these patients. Firstly, the development of low-toxicity conditioning regimens targeting sub-myeloablative busulfan levels has enabled successful transplantation, even in older patients with significant pre-existing co-morbidities ^63^, giving survival of more than 95%. Furthermore, some of the concerns about long-term sequelae of chemotherapy may be partially resolved, particularly with regard to fertility ^64, 65^. Long-term sequelae of treosulfan-containing regimens are less certain, but short-term results in survival and establishment of immune function are encouraging ^24, 25^. For many patients with PID, partial donor chimerism is sufficient to induce cure if the affected recipient cell lineage is replaced completely or partially by donor cells, although complete donor chimerism is best in some diseases ( Table 1).

**Table 1.** Examples of primary immunodeficiencies for which complete or partial donor chimerism will achieve disease cure.

Disease	Partial or complete chimerism	Donor cell lineage required
X-linked SCID	Partial	T lymphocytes
IL7Ra SCID	Partial	T lymphocytes
RAG SCID	Complete	All
Artemis SCID	Complete	All
Chronic granulomatous disease	Partial	≥10% of myeloid
CD40 ligand deficiency	Partial	≥10% of T lymphocytes
Wiskott-Aldrich syndrome	Complete	All

IL7Ra, interleukin 7 receptor alpha; RAG, recombination-activating gene; SCID, severe combined immunodeficiency.

## Transplantation of patients with no matched donor

In contrast to patients with SCID, there is usually sufficient time to seek alternative non-family adult matched donors or cord blood stem cell units from national and international registries. Owing to the following, T lymphocyte-depleted haplo-identical donors have not been widely used in non-SCID PID conditions:

• The increased risk of non-engraftment or rejection.
• Patients often harbor pre-existing viral infection. T-lymphocyte depletion of the allograft to prevent GvHD prolongs the time to immune reconstitution, thus increasing the risk of death from disseminated viral infection.
• Most non-SCID disorders can be managed in the medium term with supportive care, including prophylactic antimicrobials, immunoglobulin replacement, immunosuppressive agents, and careful nutritional and respiratory support.

New methods of depleting allografts of T lymphocytes that cause GvHD, but retaining those with an anti-viral effect, are demonstrating efficacy for patients with non-SCID PID. The most widely used technique involves depletion of CD3 ^+^ T lymphocytes bearing the αβ T-lymphocyte receptor, as well as CD19 ^+^ lymphocytes, by using magnetic bead technology. Two groups have published results with excellent outcomes of 97% survival in 37 patients with different PIDs ^65^, and 91% in 23 patients with non-malignant disease, of which 13 had PID ^66^, results which compare favorably with historic data ^56^. Although some patients developed mild GvHD, the few deaths were from viral infection. Fewer data are available for the other methods of T-lymphocyte depletion. Naïve (CD45 ^+^), rather than memory, T lymphocytes are predominantly associated with allo-reactivity and mediate GvHD ^67^. A pilot study of depletion of CD45RA ^+^ T lymphocytes, using magnetic bead technology, in five patients with combined immunodeficiencies showed promising results, with four patients engrafting and clearing viral infection within 2 months of transplantation, and no development of GvHD ^68^. Cyclophosphamide is non-toxic to pluripotent HSC but selectively toxic to recently activated lymphocytes and so administration a few days after HSCT would target newly activated previous naïve cells stimulated by recipient allo-antigens, but preserving anti-viral competence. To date, there are no published data on the infusion of replete HLA haplo-identical grafts followed by administration of cyclophosphamide after stem cell infusion for patients with PID. However, the technique has been shown to be successful for malignant conditions ^69^ and there are early reports of its use in non-malignant conditions ^70, 71^.

In conjunction with the less toxic conditioning regimens, the transplant outlook for PID patients without a good HLA-matched donor is now significantly improved. For patients with Wiskott-Aldrich syndrome, an alternative approach is the use of gene-transduced autologous cells. Initial clinical trials using retro-viral vectors resulted in insertional mutagenesis, giving rise to lymphoproliferation and leukemia ^72^. The use of lenti-viral vectors does not seem to induce *in vivo* clonal selection with vector integrations near oncogenes, and partially reverses the Wiskott-Aldrich phenotype with significant clinical effect ^73^.

## Management of post-transplant sequelae

Three major complications of HSCT are GvHD, overwhelming viral infection and sinusoidal obstruction syndrome (SOS). New developments in tackling these sequelae are beginning to reduce significant complications associated with them. T-lymphocyte depletion of donor stem cell sources and less toxic chemotherapy regimens will reduce the risk of GvHD. First-line treatment of acute GvHD is systemic corticosteroids, but for steroid-recalcitrant or steroid-resistant GvHD, treatment options that do not cause further profound immunosuppression and are not associated with significant adverse events are limited. Extracorporeal photopheresis has long been recognized as a treatment for chronic GvHD, but use in acute GvHD is now being explored ^74^. Published data have demonstrated efficacy, and with experienced operators, treatment of low-body weight (<40 kg) patients is possible. Clinical trials investigating rapid and early intervention with extracorporeal photochemotherapy (ECP) in patients with acute GvHD are in progress.

Systemic viral infections, particularly with cytomegalovirus, Epstein-Barr virus and adenovirus, remain a major cause of morbidity and mortality in all HSCT patients. Available anti-viral pharmacotherapy has limited efficacy and major associated toxicities, particularly myelosuppression and nephrotoxicity. Effective anti-viral immunity is established only when viral-specific T lymphocytes develop. Particularly in the context of T lymphocyte-depleted stem cell sources, this may take several months. Infusion of *ex vivo* expanded donor- or third party-derived T lymphocytes with activity against one or more viruses whilst excluding allo-reactive GvHD-causing T lymphocytes has been used successfully in preventing and treating viral infections following HSCT. A recent retrospective study of 36 patients with PID treated with viral-specific T lymphocytes before or after HSCT showed an overall survival of 80% with mild, self-limiting GvHD in some patients only (Michael Keller, personal communication). Previous studies have focused on the use of virus-exposed donors as a source of viral-specific T lymphocytes, but trials are now focusing on cells that have been generated from healthy donors and banked for use as “off the shelf” therapy for viral infections, which eliminates the time and expense required for custom-produced products ^75^.

SOS is a severe and potentially life-threatening complication occurring after HSCT and secondary to sinusoidal endothelial cell damage. Endothelial cell damage in other organs can lead to associated syndromes, including capillary leak syndrome, engraftment syndrome, transplant-associated microangiopathy or diffuse alveolar hemorrhage. Risk factors for the development of SOS include allogeneic HSCT, use of unrelated or HLA-mismatched donor, young age (<2 years), myeloablative conditioning, particularly when busulphan or irradiation is used, and previous or current hepatic damage. Disease-specific risk factors include hemophagocytic lymphohistiocytosis and osteopetrosis. Mortality for severe SOS with multi-organ failure is high (>80%). Numerous treatments have been used to treat SOS, including ursodiol, glutamine, vitamin E, low-molecular-weight heparin, recombinant tissue plasminogen activator, and prostaglandin E _1_. Most show dubious efficacy and are associated with significant toxicities and, in particular, hemorrhage. Supportive therapy includes early and meticulous fluid and electrolyte balance and judicious use of diuretics. Respiratory support, peritoneo-centesis and hemodialysis or hemofiltration to support renal impairment and fluid balance may be required in severe SOS. Recently, the European Medicines Agency approved defibrotide in European countries as the only curative treatment of severe SOS after HSCT. Defibrotide, a polydisperse oligonucleotide, exhibits local anti-thrombotic, anti-ischemic and anti-inflammatory properties and seems to protect endothelial cells and restore the disrupted thrombotic-fibrinolytic homeostasis. Remission from SOS and survival seem better in children than adults when receiving defibrotide ^76^. Defibrotide has also demonstrated benefit when used as prophylaxis to prevent SOS in pediatric patients, and interestingly patients receiving defibrotide also developed significantly less GvHD ^77^.

In conclusion, steady improvements in the outcome of HSCT for SCID and other PIDs mean that, for the majority of patients born with these conditions today, curative treatment is to be expected. Advances in tackling the recognized complications of HSCT have enabled survival today to approach 90%, even for patients with significant disease-related sequelae. As new diseases are described, the challenge is to determine the best therapeutic option, but HSCT is a realistic treatment for many patients, including adults with late-diagnosed or late-onset disease. Important questions remain, however; the three most pressing are how to achieve earlier diagnosis, how to develop non-toxic conditioning regimens to achieve durable and sustained immune reconstitution, and an evaluation of the long-term outcomes of HSCT for these conditions, including immune function and general and psychological health. Continued collaborations between physicians caring for these patients and scientific societies dedicated to the study of diseases and their treatments are likely to further these aspirations. As we learn more about the biology of these conditions and their treatments, the information may also benefit patients receiving HSCT for other diseases as well as those taking part in gene therapy trials and patients with autoimmune diseases or those receiving solid organ transplants.

## Abbreviations

ADA, adenosine deaminase; GvHD, graft-versus-host disease; HLA, human leukocyte antigen; HSC(T), hematopoietic stem cell (transplantation); NK, natural killer; PEG-ADA, polyethylene-glycosylated adenosine deaminase; PGM3, phosphoglucomutase 3; PID, primary immunodeficiency; SCID, severe combined immunodeficiency; SOS, sinusoidal obstruction syndrome; TREC, T-cell receptor excision circle.
